# Supplementary figures and images for: Fexinidazole results in specific effects on DNA synthesis and DNA damage in the African trypanosome
Source: PLoS Negl Trop Dis. 2025 Oct 24;19(10):e0013647. doi: 10.1371/journal.pntd.0013647 (PMC12574827; doi:10.1371/journal.pntd.0013647)

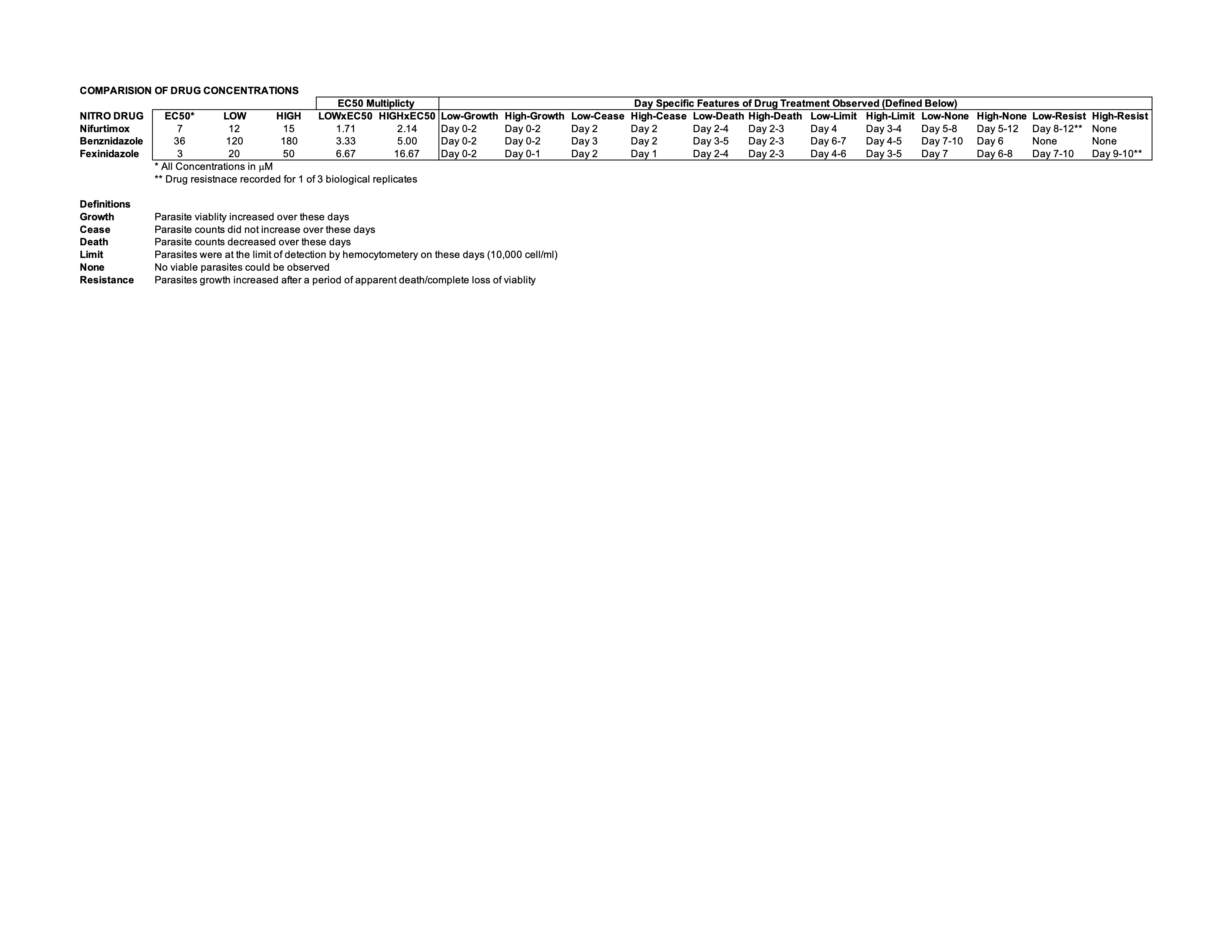

Supplement: S1 Table — (TIFF) [file pntd.0013647.s001.tiff]

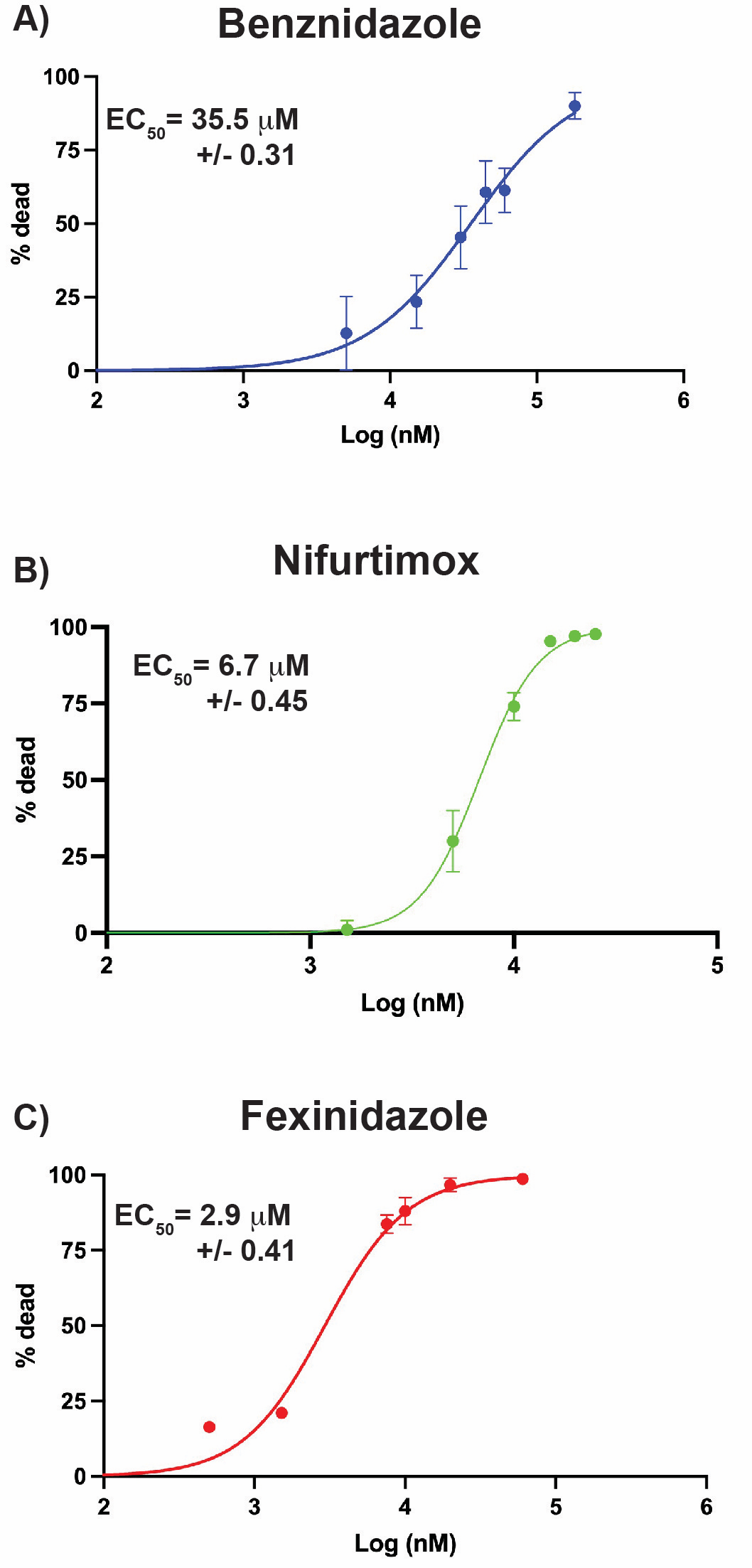

Supplement: S1 Fig — Cell viability assays were conducted using AlamarBlue alongside Puromycin control for 100% cell death to measure the EC50 of Benznidazole (A), Nifurtimox (B), and Fexinidazole (C). Values show drug concentrations (μM) and standard deviations resulting parasite death. (TIFF) [file pntd.0013647.s008.tiff]

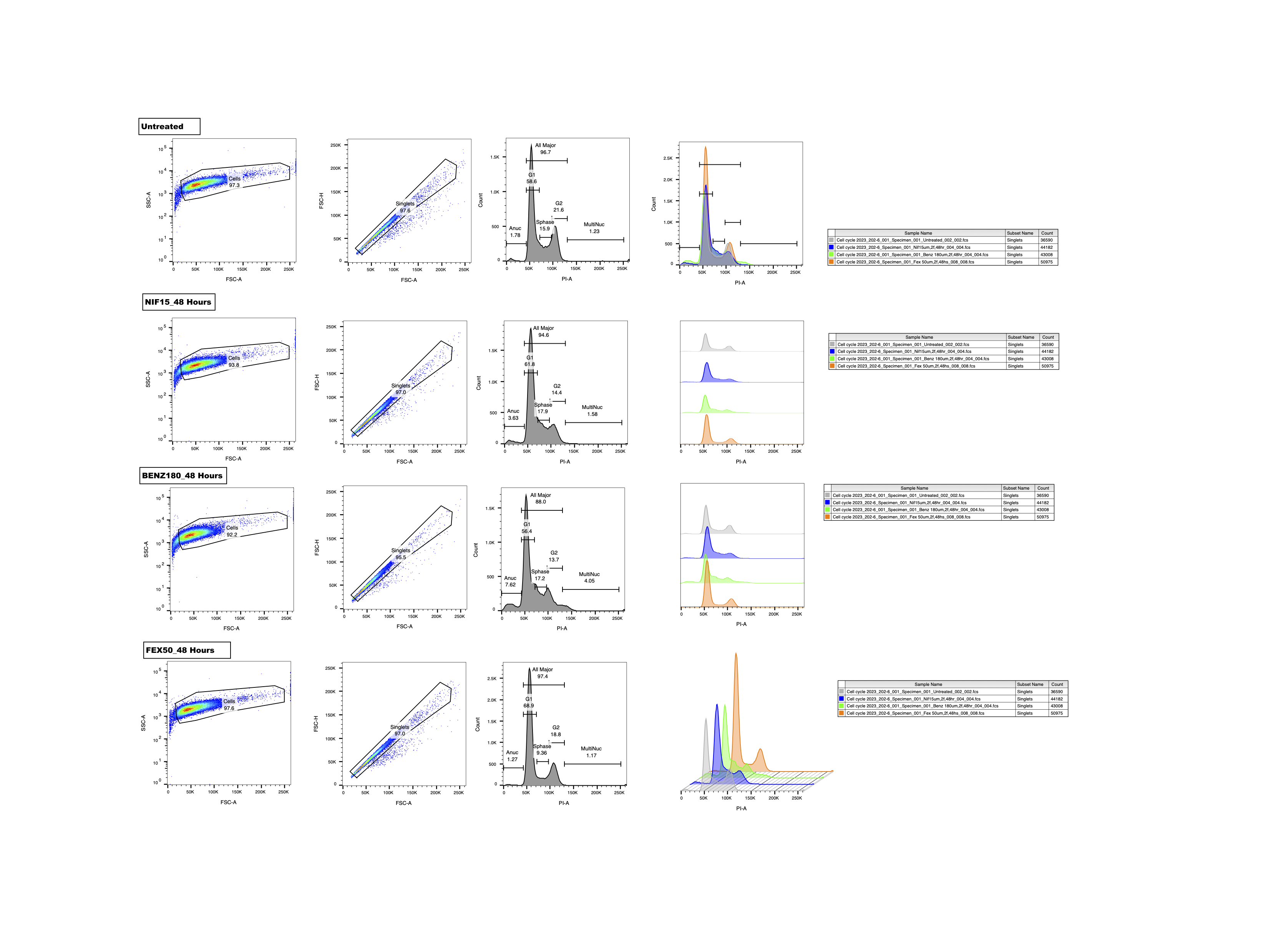

Supplement: S2 Fig — Representative data is shown for cell cycle gating for untreated and HIGH drug concentrations after 48 hours for a single replicate (All replicate data can be found in S2 Data. First Row displays the “Cell Gate”, isolation of the cell associated events. Second Row displays “Doublet Discrimination”, removal of doublet cells from data analysis such that only singlets are evaluated for cell cycle events. Third Row shows cell cycle gates for each population analyzed: Anucleated, G1, S phase, G2, and Multinucleated cell populations were quantified based on these gates. The fourth and final row shows alternative overlay visualizations of the resulting gated cell cycle data. (TIFF) [file pntd.0013647.s009.tiff]

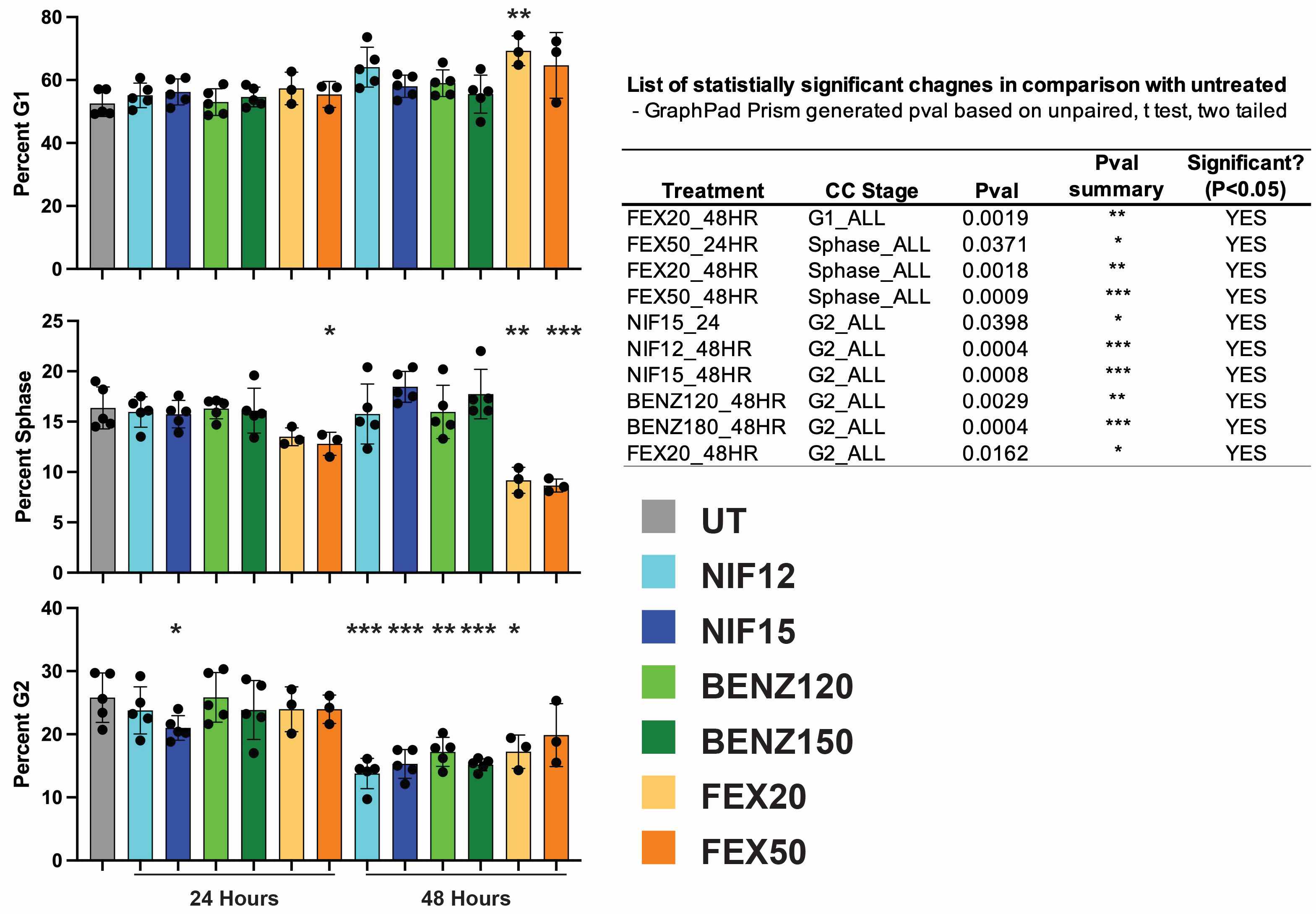

Supplement: S3 Fig — Drug effects on G1 (top), S phase (middle), and G2 are shown for nifurtimox (light blue and dark blue), benznidazole (light green and dark green), and fexinidazole (yellow and orange) over 24 hours (Left) or 48 hours (Right) of drug treatment in comparison with untreated (UT) control. Cell cycle population changes of statistical significance are shown on the table and marked with asterisks according to their magnitude of significance, P values * < 0.05, ** < 0.005, *** < 0.0005. (TIFF) [file pntd.0013647.s010.tiff]

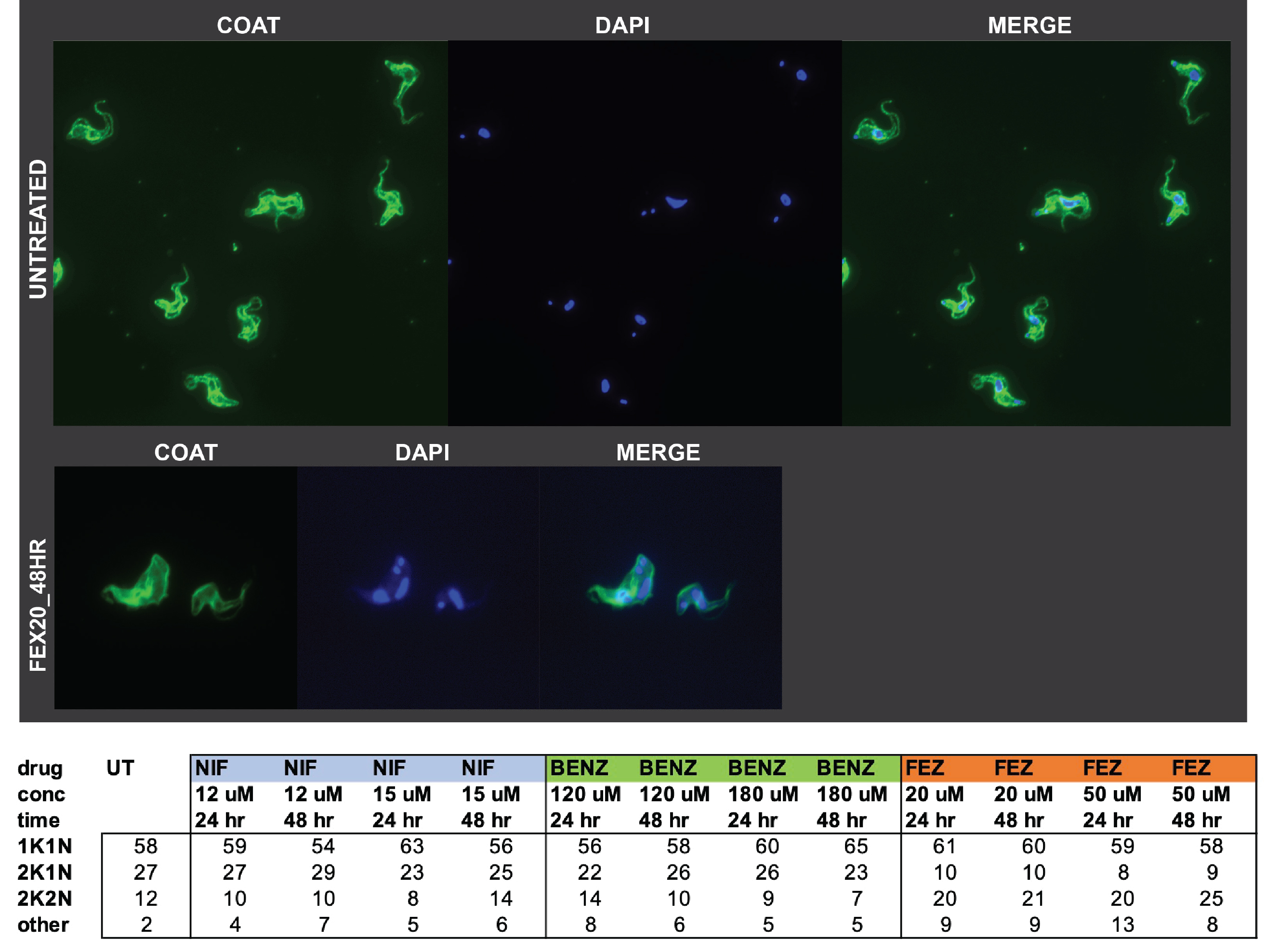

Supplement: S4 Fig — The distribution of kinetoplasts and nuclei per parasite are shown by immunofluorescence microscopy using DAPI (DNA content staining) and anti-VSG-2-Alexa488 conjugated antibody for the parasite cell surface. Untreated presents a sample of the normal distribution of 1K1N (G1) cells and 2K1N (G2) cells in normal parasite populations. Fexinidazole treated (20 μM at 48 hours) parasites illustrated “other” cells types as nuclear anomalies arising from fexinidazole treatment. Bottom – Table of percent populations arising from all conditions, treated and untreated. (TIFF) [file pntd.0013647.s011.tiff]

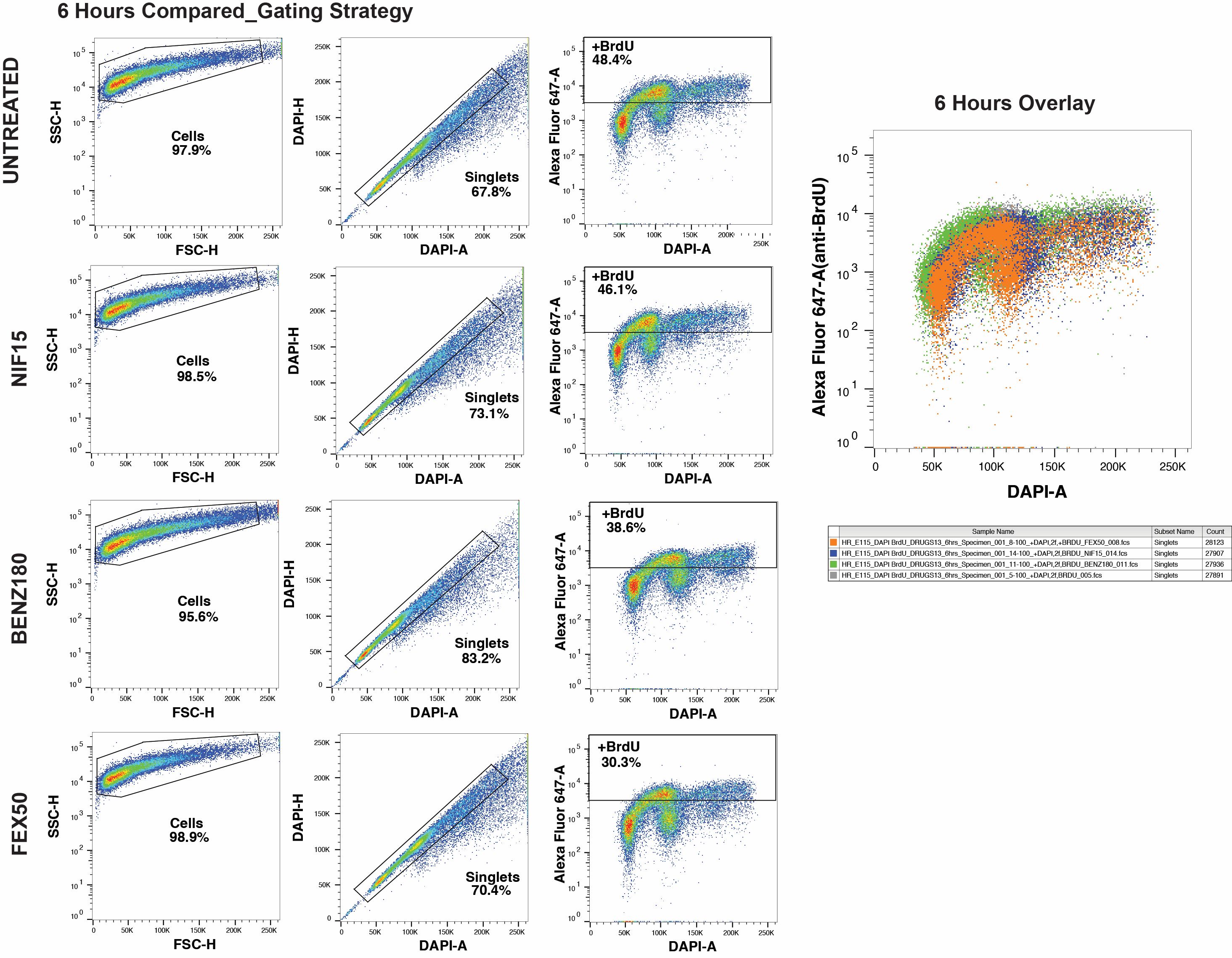

Supplement: S5 Fig — Representative data is shown for BrdU incorporation assay gating for untreated and HIGH drug concentrations after 6 hours for a single replicate (All replicate data can be found in S3 Data. First Row displays the “Cell Gate”, isolation of the cell associated events. Second Row displays “Doublet Discrimination”, removal of doublet cells from data analysis such that only singlets are evaluated for cell cycle events. Third Row shows quantification of the DNA synthesizing population based on staining with the anti-BrdU antibody conjugated with Alexa-647 (y-axis) vs. DAPI DNA content staining (x-axis). The + BrdU population gate shown is the source of percent DNA synthesis used in the analyses of Fig 4. On the right a representative overlay of the data is shown. (TIFF) [file pntd.0013647.s012.tiff]

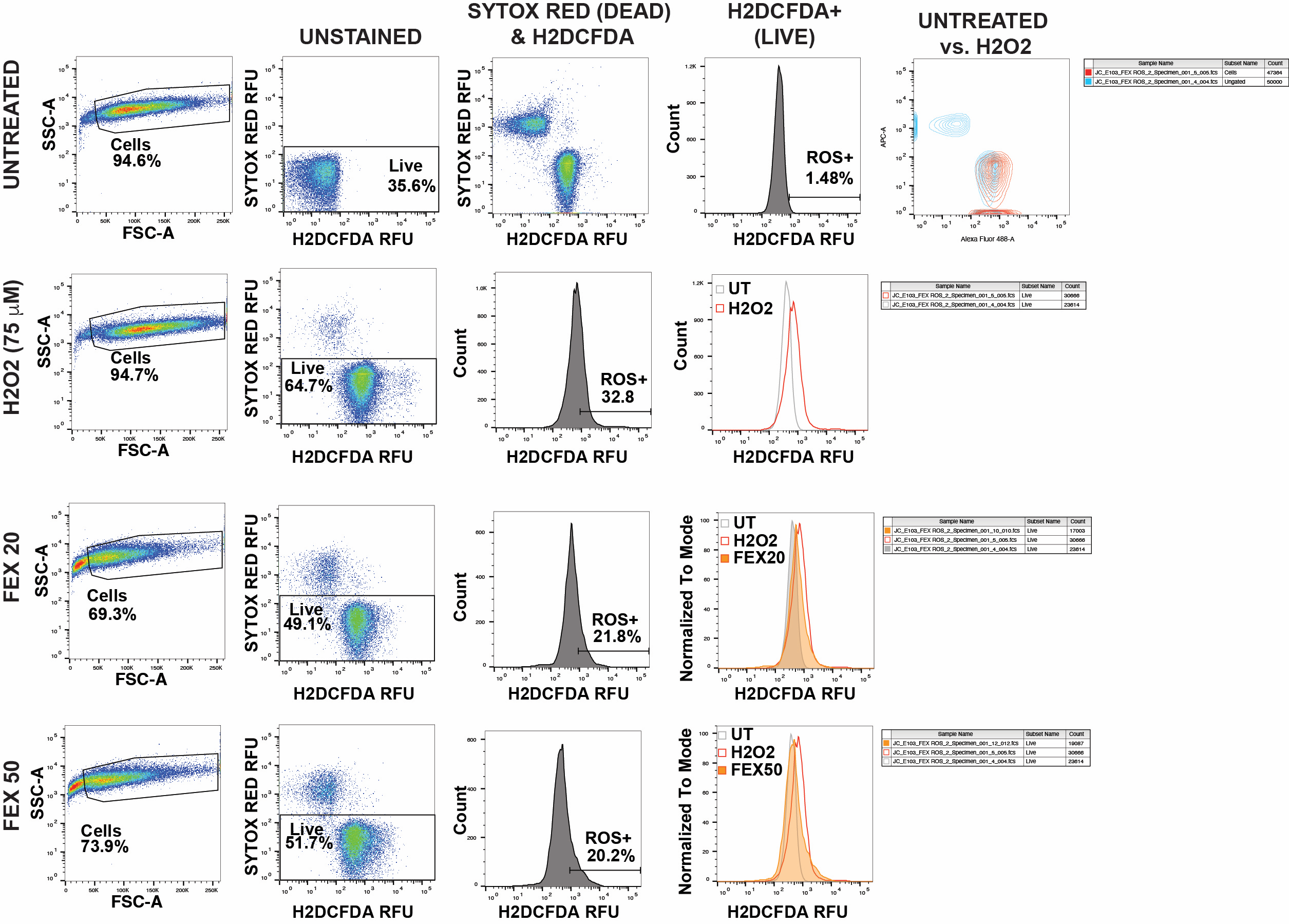

Supplement: S6 Fig — Representative data is shown analysis of ROS by H2DCFDA staining showing a single replicate of Untreated, H2O2 positive control treatment, FEX μ20, and FEX 50 μM. First Row displays the “Cell Gate”, isolation of the cell associated events. Second Row displays live vs. dead discrimination using SYTOX RED fluorescence on the y-axis. Live cells are gated (with dead cells removed) and then used to evaluate H2DCFDA under shown conditions. The “ROS+” percentage was gated based on H2DCFDA stained but Untreated cells (that is not ROS stressed). This gate was then applied to all treated samples. Overlays are shown of H2DCFDA histograms for untreated and treated conditions. (TIFF) [file pntd.0013647.s013.tiff]

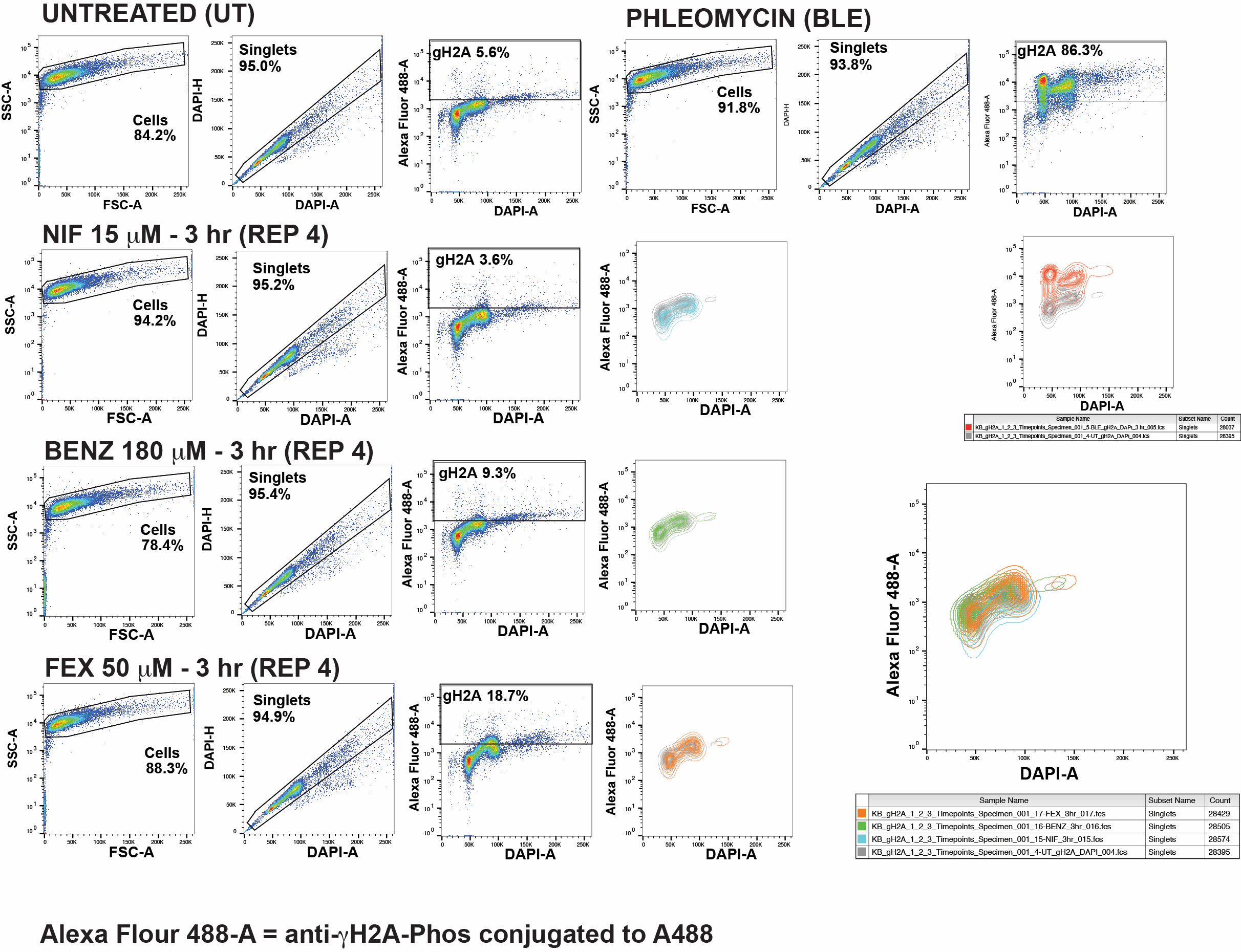

Supplement: S7 Fig — Representative data is shown analysis of DNA damage by γH2A-Phosphorylation using a T. brucei specific anti-γH2A-Phos antibody fused to Alexa-488. Gating shows a single replicate of Untreated and each drug at their HIGH concentrations after 3 hours. First Row displays the “Cell Gate”, isolation of the cell associated events. Second Row displays Doublet discrimination based on DAPI staining for the identification and subsequent analysis of the Singlet population. In the third row singlets are plotted against anti-γH2A-Phos-A488 (y-axis) for DNA damage vs. DAPI content stating (x-axis) for evaluation of cell cycle stge. Overlays are shown for each treatment compared to untreated by countour plot as well as all drug treatments overlayed. (TIFF) [file pntd.0013647.s014.tiff]

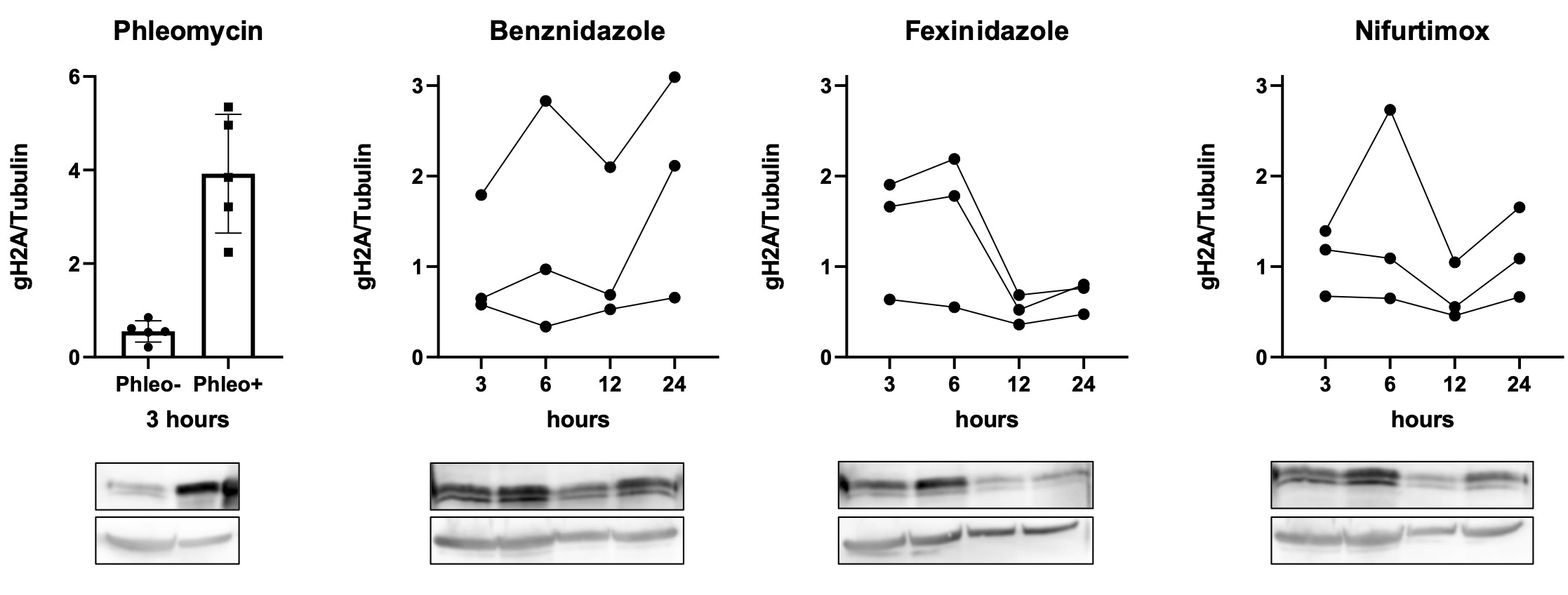

Supplement: S8 Fig — Quantitation of anti-γH2A-Phos western blots normalized to anti-Tubulin control (bottom lane) for benznidazole (180 μM), fexinidazole (50 μM), and nifurtimox (15 μM) over 3, 6, 12, and 24 hours in comparison with untreated (shown as Phleo-) and phleomycin treated positive control (Phleo+). (TIFF) [file pntd.0013647.s015.tiff]

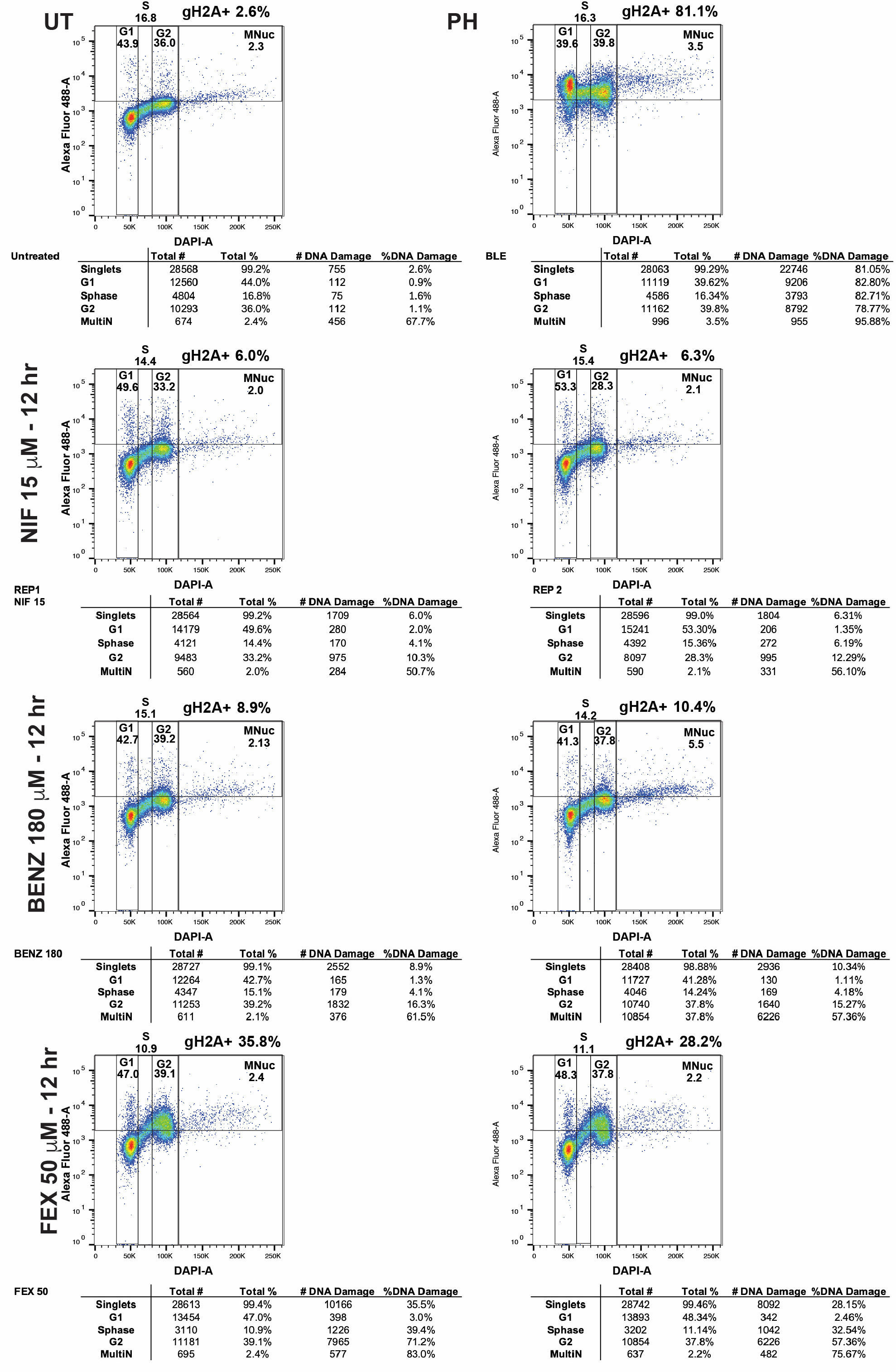

Supplement: S9 Fig — Representative data from UT, BLE treated, and two replicates of each NIF 15, BENZ 180, and FEX 50 after 12 hours of treatment are shown. Data shown on the flow cytometry plot include – percent total gH2A+ (DNA damaged cells) shown above the plot, percent of events gated in G1, S, and G2. Tables below each plot show the number of total events and DNA damage specific events arising in each stage of cell cycle analyzed. The percent of DNA damage from each stage of cell cycle is derived as the number of gH2A+ events divided by the total number of events in that cell cycle stage. Full data for all conditions and replicates can be found in S6 Data. (TIFF) [file pntd.0013647.s016.tiff]
