# Supplementary material for: Fexinidazole results in specific effects on DNA synthesis and DNA damage in the African trypanosome
Source: PLoS Negl Trop Dis. 2025 Oct 24;19(10):e0013647. doi: 10.1371/journal.pntd.0013647 (PMC12574827; doi:10.1371/journal.pntd.0013647)
Supplement: Attachment — Submitted filename: FEX MAN_NTD_Response FINAL.docx [file pntd.0013647.s018.docx]

PNTD-D-25-00887
Fexinidazole results in specific effects on DNA synthesis and DNA damage in the African trypanosome
PLOS Neglected Tropical Diseases

Dear Editors and Reviewers,

Thank you for taking the time to comprehensively consider the data and implications of our study entitled, “Fexinidazole results in specific effects on DNA synthesis and DNA damage in the African trypanosome.” We appreciate that you acknowledge the timely significance of the work, our use of comparative design between drugs and their cytotoxic outcomes, and that this work has the potential to inform drug-induced DNA damage pathways in protozoan parasites. We also appreciate the criticisms of the work, which primarily focused on clarification of the statistical relevance of our findings and that our discoveries are more observational in nature than mechanistic. Based on your comments, we have sought to prepare a significantly improved manuscript by way of a ***major revision*** that is focused on reinforcing the complete transparency of our data, the associated statistics, and the conservative presentation of their mechanistic implications (or lack thereof). We thank Reviewer #2 for stating that, “**None of the above issues warrant rejection or invalidate the conclusions but are important for enhancing scientific communication and reproducibility.”** Thus, this revision was aimed to improve the quality, clarity, and statistical robustness of the manuscript. As this this study is the first to delineate the trypanocidal effects of fexinidazole, we believe that it is an important contribution to the field of neglected tropical diseases and the drugs that treat them.

This response will be structured in the following manner:

1^st^ – Outline all changes to figures, supplements, figure legends, and methods that constitute a major revision in response to the reviewers’ comments.

2^nd^ – Specific commentary on mechanism and changes made in responses to Reviewer 2.

3^rd^ – Additional line by line comments from reviewers will be addressed in order along with associated manuscript changes.

***We appreciate the consideration of PLOS NTD and hope that you will find the following changes sufficient to accept our discoveries for publication in your esteemed journal.***

**Major Revision Changes to Figures, Supplements, Figure Legends, and Methods Sections**

The focus of this revision section was to increase clarity regarding our applied statistics, expand our statistical comparisons, and improve data availability by adding supplements for all gating strategies and that include all of the raw counts associated with each relevant experiment.

**Notes on the revised manuscript:**

- Changes to text in the manuscript will be shown in blue.

- Sections that have been changed, will be highlighted herein in blue.

- Line references refer to the marked-up copy. The marked-up copy retains figures as inline text at the end of the document with figure legends below each figure.

- The clean manuscript copy has no notations to changed made in review.

- In keeping with PLOS NTD guidelines the ***clean copy has figure legends immediately following the associated results section***.

* This results in differences in line numbers between the marked-up copy and the clean copy.

Methods:

A new Methods section was added entitled “Statistical analysis of flow cytometry data” was added to NEW Lines 265-276, which was the in direct response to Reviewer #1 and #3. This section delineates the process of flow cytometry data analysis, all relevant software and versions used, and the basis for all statistical analyses shown in associated figures and supplements.

Modifications made to Figures, Legends, and Supplements:

***FIGURE 1*** – Figure 1 was modified so that each graph shown is based on 3 biological replicates from each drug treatment condition. In addition, error bars were removed from spontaneous resistant populations in FIG. 2B NIF 12 and FEX 50, because resistance only arose in 1 replicate of each condition. This information is now included in the figure legend.

Supplemental data –

NEW Table SUP. 2 (.pdf): Comparison of Drug Concentrations (pdf)

In response to Rev 3 suggestion, table compares the EC50 of each drug to the concentrations used in this study along with when each concentration results in parasite death related outcomes.

NEW Table SUP. 3 (.xlxs): RAW CELL COUNTS FROM FIG 1

All raw hemocytometry cell counts from all experiments and replicates in FIG. 1 are shown.

The new supplemental tables are now referenced in the Results lines 305-307, which now includes further clarification of our parameters in selecting the “LOW” and “HIGH” concentrations used.

***FIGURE 2*** – In response to Rev 1 comment, we expanded our statistical comparison beyond untreated vs. drug to include fexinidazole vs. both nifurtimox and benznidazole for S phase populations. To avoid confusion, we also eliminated the violin plots from FIG. 2. The graphs in FIG. 2B now show the statistical comparison of S phase populations of FEX in comparison with UT, NIF, and BENZ after 24 and 48 hours of drug treatment. Significance is reported based on Pvalue as described in the NEW methods section.

The text of the associated results sections has been modified as follows:

***Results*** sections from lines 327 & 336-337 have been modified to discuss the newly generated supplements and associated statistical analyses found in Figure 2 and supporting supplements 4-6.

***Figure legend 2*** has been modified to reflect the changes in data shown.

NEW SUP. 4 (.jpg): Cell cycle gating strategy.

NEW SUP. 5 (.xlxs): All cell cycle flow cytometry data raw counts exported from FlowJo and processed data used in statistical analyses present in excel file tabs.

SUP. 6 (.jpg): Previously SUP. 2, Graphical and statistical analysis of drug treatments in comparison with UT.

***FIGURE 3*** – No Changes.

***FIGURE 4 –*** No changes to manuscript figure. Two new supplements added for BrdU incorporation assay flow cytometry gating strategy and source data for raw counts and statistics.

NEW SUP. 8 (.jpg): Cell cycle gating strategy for DNA synthesis quantification by BrdU incorporation assay.

NEW SUP. 9 (.xlxs): All raw counts from Flowjo gating exported and replicates and statistical data shown in figure 4.

***FIGURE 5 –*** No changes to manuscript figure. Two new supplements added for ROS analysis by H2DCFDA a flow cytometry gating strategy and source data for raw counts and statistics.

NEW SUP. 10: Figure (.jpg) – Gating strategy for H2DCFDA analysis of ROS by flow cytometry

NEW SUP. 11: Tables (.xlxs) – All flow cytometry data from H2DCFDA analysis of ROS.

***FIGURE 6 –*** In response to Reviewer 3 two changes were made to FIG. 6 of the manuscript. 1) We agree that the Overlay data is better highlighted using contour plots and have made this change and 2) We have removed the 24 hour timepoint from the associated γH2A-Phos western blot analysis to avoid confusion.

NEW SUP. 12: Figure (.jpg) – Gating strategy for DNA Damage analysis by γH2A-Phos by flow cytometry

NEW SUP. 13: Tables (.xlxs) – All flow cytometry data used in γH2A-Phos analysis of total DNA damage.

* Note: A comparison of FEX vs. Other Drugs was added and there was a statistically significant difference between FEX and drugs compared at all by one timepoint. While this relevant information was added to the manuscript text in lines 481-483 no additional figure was made to display this information.

***FIGURE 7 –*** Based on the reviewers’ comments we modified FIG. 7 to increase clarity regarding important aspects of this discovery in the following ways: 1) FIG. 7A now shows gating and representative data for UT and each of the drug treatment conditions. G1, S phase, and G2. Along with the total %DNA damage in red. 2) FIG. 7B Graphs of DNA damage vs. time now show the statistical significance of the difference between FEX and other drugs at each time point in each cell cycle stage. 3) FIG. 7C Now highlights the graphical analysis of the percent of DNA damage per cell cycle stage for each drug and timepoint. While eliminating the previous figure that highlighted the effects of phleomycin to show cell cycle gates. 3

NEW SUP. 15: Figure (.jpg) – Gating strategy for DNA Damage per cell cycle stage by γH2A-Phos and DAPI staining

NEW SUP. 16: Tables (.xlxs) – All raw exported data and analyzed flow data associated with determining the percent of DNA damage per cell cycle stage.

*This concludes the major revision of the figures and data presentation in this manuscript. While only minor changes were made to the main body figures, an* ***additional 12 Data Supplements*** *were generated to increase the statistical clarity and data availability of this study.*

**PART 2 – Response to Reviewer #2 Concerns Regarding Assertion of Mechanism**

Comments from Reviewer #2 in black 10 pt arial, responses in 12 pt, with changes in blue.

Reviewer #2: This manuscript investigates the cytotoxic effects of fexinidazole, a clinically approved oral nitroaromatic drug, in Trypanosoma brucei, and compares its activity with that of nifurtimox and benznidazole. The authors evaluate the effects of these compounds on cell cycle progression, DNA synthesis, DNA damage (via γH2A phosphorylation), and ROS production. This work aims to dissect whether fexinidazole exerts distinct cellular effects that may inform its mechanism of action and distinguish it from related nitroaromatic agents.

We thank Rev #2 for this summary and the accuracy of the final statement, as we sought to dissect the observed cellular effects of fexinidazole that may ***inform its mechanism of action*** in comparison with related drugs.

Weaknesses and Major Limitations:
Despite its strengths, the manuscript falls short of providing definitive mechanistic insight and currently overinterprets several findings:

We thank Rev #2 for their thoughtful insights and generally agree that the data presented are largely observational and did not intend to assert demonstration of mechanism in this manuscript. To further clarify and appropriately limit the interpretations of our data we have made the following changes:

**Abstract**

“Deconvolving the relationship between anti-parasitic drugs and the molecular basis of their cytotoxic outcomes will support future mechanistic understanding and enable improved drug design.”

**Author Summary (New)**

In the newly added author summary includes the line, “While the precise molecular mechanisms underlying these outcomes remain to be elucidated, these findings provide critical new insights into fexinidazole’s trypanocidal activity.” We hope that this line adds clarity to the observational nature of the data generated in this study.

**Intro**

Line 167-169: Now read - Data generated from the γH2A-phosphorylation show that fexinidazole treatment can result in significant levels of DNA damage in S phase and G_2_ after as little as 3 hours of treatment, suggesting that DNA damage formation may be a key factor in fexinidazole’s trypanocidal activity

Line 169: The mechanistic implications of fexinidazole’s cytotoxic effects will be discussed.

The word “putative” has been added-

“The putative mechanistic implications of fexinidazole’s cytotoxic effects will be discussed.”

**Results**

Previous Line 390-392: These data strongly suggest that the decrease in DNA synthesis observed during FEX treatment is likely associated with the drug’s capacity to damage DNA (see putative models in FIG. 8).

NEW 521-523 – One potential implication of these data is that DNA damage arising during FEX treatment may contribute to the FEX induced DNA synthesis defect reported herein (see putative models in FIG. 8).

Previous Line 395-397: Elucidating precisely how fexinidazole causes DNA damage and the interplay between the observed DNA synthesis and DNA damage phenotypes reported here will be the subject of future mechanistic studies.

We apologize as it was not our intention to specify causality in this phrase. We have changed to clarify the difference between the observation that FEX treatment results in DNA and not a statement of causality that was not proven:

NEW 524 - Elucidating precisely how fexinidazole treatment results in DNA damage and the interplay between the observed DNA synthesis defect and DNA damage phenotypes reported here will be the subject of future mechanistic studies.

Inferred causality is not experimentally demonstrated.
The authors propose that fexinidazole-induced DNA damage precedes and drives DNA synthesis inhibition and ROS formation. However, this sequence is inferred based on asynchronous measurements across different timepoints and lacks direct experimental validation. Without synchronized assays or causal perturbations, this interpretation remains speculative.
It was not our intention to propose this specific order of events in the “causality” of FEX-induced cell death. Throughout the manuscript we have removed statements of implied causality to favor more discrete scientific terminology, such as “FEX treatment results in”.

To further avoid confusion regarding FIG. 8, we have made the following changes. The goal is to present two contrasting putative models to explain the data presented, without asserting unsubstantiated molecular mechanisms.

1) Added “?” next to the arrows between FEX and the outcomes of ROS stress or DNA damage in indicate that the chemical causality of this effect is unknown.

1a) This is now noted in the figure legend in the following text: by an unknown molecular mechanism denoted with “**?**”

2) We changed the name of the figure to remove the word mechanism.

3) The new figure name is “**Contrasting putative models of fexinidazole induced cytotoxicity”.**

4) The figure legend now specifies the contrasting comparison of two models that could explain the observed outcomes.

5) The discussion is now framed in presenting contrasting models in figure 8 from lines 552-557.

Nature of DNA damage is not characterized.
The exclusive reliance on γH2A phosphorylation does not distinguish between types of DNA lesions (e.g., strand breaks vs. oxidative adducts). This limits the conclusions that can be drawn about the underlying mechanism.

This is correct. We look forward to addressing these questions in more detailed mechanistic future studies. We hope we have clarified above that we were not seeking to draw or publish conclusions about underlying mechanism.

Drug dosing strategy lacks standardization.
The use of differing multiples of EC50 across drugs (e.g., 6×EC50 for fexinidazole vs. 2×EC50 for benznidazole) compromises the comparability of phenotypic effects and may exaggerate fexinidazole’s distinctiveness.
We acknowledge that the mode of standardization is unusual. This is the entire subject of figure 1 (and now SUP. 2). For the purposes of this study, it was essential to establish a standardization method based on cell growth and death dynamics. If we had utilized a standard multiplicity of EC50, as others have, the timelines of continued growth from day 0-2, cessation of growth from day 2-3, at the limit of detection from 3-6, no observable parasites from day 5-7 would not have been consistent. Our stated goal was to be able to conduct compliable assay over the first 24-48 hours and would not have been possible with a standard based on EC50 alone.

This is the subject of 312-315: “While imperfect, the LOW & HIGH drug concentrations empirically established here provided the most comparable drug concentrations of NIF, BENZ, and FEX for evaluating cytology phenotypes between 0 and 48 hours of treatment and will be used throughout this study.”

Additional information on this can now be found in lines 305-307 and refer to the new generated SUP. 2, which provides a direct comparison of the EC50 of each drug and the concentrations used in this study along with their comparative growth and death outcomes.

No investigation of DNA repair or downstream pathways.
The authors mention the potential involvement of oxidative DNA repair mechanisms (e.g., 8-oxoG glycosylase) but do not experimentally assess whether these are activated or impaired during fexinidazole exposure.

Correct, this is mentioned in the discussion and will be the subject of future studies arising from this work.

Novelty and Significance:
The manuscript provides new comparative phenotypic data on three nitroaromatic drugs in T. brucei and identifies a potentially distinctive inhibitory effect of fexinidazole on DNA synthesis. The implementation of flow cytometry to measure DNA damage across cell cycle stages is technically valuable and potentially broadly applicable.

We thank Rev #2 for their acknowledgement that our data provide new data and identify potentially distinctive aspects of FEX effects. As well as the technical advances to DNA damage analysis that we believe will be broadly applicable.

However, to achieve the level of mechanistic novelty necessary for publication in PLOS Neglected Tropical Diseases, the manuscript must go beyond observational data and test the proposed relationships between phenotypes more rigorously.
We agree that PLOS NTD is a highly esteemed journal and think our data is an excellent fit for both its mission statement and scope. Respectfully, we are not aware of a bar for publication in PLOS NTD that requires demonstration of mechanism (https://journals.plos.org/plosone/s/criteria-for-publication). The originality and importance of the work are both paramount in that ***no cytotoxicity studies have ever been published for the clinically utilized drug fexinidazole***. In addition, our insights into nitroaromatic drug cytotoxicity will be of interest to the broader research community. Our methodology, which was highly rigorous, is now more so with the additional statistical analyses and raw data included in this revision. We are profoundly respectful regarding any overstatement of the mechanistic basis of our findings and seek to absolutely adhere to presenting only justified and evidence-based conclusions. Finally, the ethical handling of our research and data presentation is of paramount importance in this and all studies arising from our group.

We hope that the increased rigor of the data analysis associated with the additional supplements and statistical analyses further satisfy Rev #2.

New Experiments Required for Acceptance (Major Revision):
To support the authors’ central hypothesis and improve mechanistic depth, the following experiments or analyses are strongly recommended:

The manuscript under consideration did not assert a central hypothesis or assert mechanism. In our own words we:

- …evaluate the trypanocidal outcomes of fexinidazole treatment in comparison with nifurtimox and benznidazole.

- Our findings show that, while nifurtimox and benznidazole have somewhat similar cytotoxic outcomes, fexinidazole treatment results in a profound and specific inhibition of DNA synthesis.

- Data generated from the γH2A-phosphorylation demonstrated that treatment with fexinidazole can specifically induce DNA damage in S phase and G_2_ after as little as 3 hours of treatment, which suggests that this effect may initiate fexinidazole’s trypanocidal activity.

- The putative mechanistic implications of fexinidazole’s cytotoxic effects will be discussed.

Previously the manuscript mentioned a hypothesis in the final lines for future studies:

“The putative pathways of drug induced DNA synthesis inhibition were well supported by our findings and provide new testable hypotheses to further elucidate anti-trypanosome drug mechanisms of action.”

However, upon further consideration and review of the PLOS NTD review criteria we have decided to clarify the purpose of the research conducted in the following, new lines 158-160: Here we investigated the trypanocidal outcomes of fexinidazole in comparison with nifurtimox and benznidazole while evaluating the proposal that nitroaromatic drugs kill trypanosomes through ROS activation of DNA damage.

We hope that this language framed at evaluating and existing proposal, rather than demonstrating mechanism will be acceptable to Rev #2.

We agree whole-heartedly with Rev #2 that our observational data provide excellent foundational observations for future hypothesis driven mechanistic studies. The following comments will be taken into consideration while planning those investigations:
Clarify the temporal relationship between DNA damage, ROS formation, and DNA synthesis inhibition:
Improve ROS detection sensitivity at earlier timepoints (e.g., 3–6 h) using more sensitive probes or alternative assays.
Optionally, use synchronized cell populations to track phenotypes more precisely through the cell cycle.
Characterize the nature of DNA damage:
Use comet assay, γH2A foci visualization, or biochemical approaches (e.g., LC-MS for DNA adducts) to determine whether damage is oxidative, alkylative, or strand break-associated.
Standardize drug dosing:
Compare drugs at equitoxic doses (e.g., IC90 or AUC-equivalent) or provide dose-response curves that justify the specific concentrations used for each drug.
Investigate DNA repair pathways:
Analyze expression or activity of candidate DNA repair genes (e.g., glycosylases, polymerases) in fexinidazole-treated vs. untreated cells, or assess parasite viability in DNA repair-deficient strains.
If these experiments cannot be completed within the revision timeline, the authors must significantly temper their conclusions and explicitly frame the mechanistic model as speculative.

We hope that the noted manuscript modifications now satisfy Rev 2 and that our models are presented as sufficiently speculative.

Scholarship and Ethical Standards:
The manuscript appears to meet scientific and ethical standards. No concerns were identified regarding research misconduct, dual publication, or data transparency. The data availability statement should be updated with links to raw data (e.g., flow cytometry files) upon revision to support reproducibility.

As we have been unable to identify a data repository for raw flow cytometry data availability, we have made the information available in the supplementary data in the following tables:

**Supplemental Data 5: Tables (.xlxs) – All flow cytometry data from cell cycle analyses**

**Supplemental Data 9: Tables (.xlxs) – All flow cytometry data from BrdU incorporation**

**Supplemental Data 11: Tables (.xlxs) – All flow cytometry data from H2DCFDA analysis**

**Supplemental Data 12: Tables (.xlxs) – All flow cytometry data from γH2A-Phosphorylation**

**Supplemental Data 16: Tables (.xlxs) – All flow cytometry data from γH2A-Phos**

Final Summary:
This study addresses an important clinical agent and provides promising comparative data. However, in its current form, the manuscript falls short of its mechanistic ambition and would benefit from deeper experimental support and more cautious interpretation. I recommend Major Revision, with the expectation that the authors either (1) perform key mechanistic experiments or (2) revise their claims to align with the descriptive nature of the data.

We again thank Rev 2 for acknowledging the importance of the study and data comparisons. We hope that we have clarified that it was not our intention to assert causality or mechanism and that we have sufficiently revised the manuscript to clarify the descriptive nature of the work. It is our view that this study and its findings are an essential step toward deeper understanding of mechanism in future works.

**PART 3 – Additional Responses to Reviewers’ Line by Line Changes**

**Response to Reviewer 1**

We thank Reviewer 1 for their careful consideration of our manuscript and acknowledgement that the detailed analysis of fexinidazole is “beneficial” as it becomes an important option in the treatment of African trypanosomiasis. Here we seek to thoroughly respond to each comment from Reviewer 1 to further support their confidence in the importance of our study and our findings.

Reviewer 1 – “…their conclusion in such the difference largely depends on impressions but not statistical analyses, and the authors need to show outcomes of statistical analyses not only in comparison of a treatment group with the control group but also among the treatment groups.”

- Additional statistical analyses of the cell cycle data of FIG. 2B to further demonstrate that the decrease in the S phase population is specific to fexinidazole. The statistical significance of the decrease in S phase can be seen in comparison with UT at 24 and 48 hours, with nifurtimox at both times and concentrations, and in all but one comparison with benznidazole. The specific Pvals for all comparisons are shown in SUP. 5 on the tab labeled “CC_Sig Pval”.

Reviewer 1 – “What is a doubling time for this parasite? According to the growth speed and as the authors state that they used BSF, the doubling time should be short, around 6 hours. This means that even during 24 hours of cultivation, the parasites undergo multiple cell cycles. A simple question is whether it is possible to have S-phase affected while still having G2-phase not much affected. Figure 3 says that FEX decreased 2K1N but increased 2K2N; where these increased 2K2N from?”

- The doubling time for bloodstream form T. brucei is between, 6.5 and 8 hours. Thus, it is correct that our cultures are asynchronous and double multiple 3-4 times during a 24 hour experiment. Regarding the decrease in S phase, represented as 2K1N, and the corresponding increase in G2, 2K2N, this is likely because G2 is undergoing a cell cycle stall such that G2 is unproductive and does not result in cytokinesis. It is not accurate to say that G2 is “not much affected”, based on our data, it is likely that cells that make it into G2 have highly damaged DNA and do not successfully complete cytokinesis. We know that at later time points the pile up in G2 and Anucleated populations increase dramatically. This is likely because of asymmetric division resulting in the formation of an anucleated daughter and 2N like parental cell. This is now addressed in greater detail in the results lines 374-377 and discussion lines 558-566.

Reviewer 1 – Also, the reviewer experienced difficulty in interpreting data discrepancy between Figures 2 and 3. Figure 2 says that FEX treatment resulted in the decrease in G2 phase as the other drugs did. On the other hand, Figure 3 says FEX treatment increased 2K2N. It would be nice if the authors can address this point as well.

- DNA content staining by propidium iodide has some inherent differences with number of nuclei and kinetoplast counted per cell using microscopic methods. We do not report a statistically significant decrease in G2 for most fexinidazole treatment conditions (only low fex 48 hours, SUP. 6). The fact that the change in the number of nuclei goes up following fexinidazole treatment where the G2 population by flow cytometry is slightly reduced may arise from the DNA content of G2 nuclei being less than normal G2 cells. It is intriguing to consider the state of the nuclear DNA in the FEX treated G2 cells and will be the subject of future studies, which is somewhat visible in SUP. 7.

Reviewer 1 – “L265: ‘Treatment with either NIF or BENZ resulted in a progressive decrease in the 2K2N population over time and concentration, which corresponds with the loss of G2 cells observed by flow cytometry (FIG. 3B).’ Not sure if this interpretation is correct. For example, NIF 48 h has 14% 2K2N (G2), which is higher than that of UT.”

- In fact, UT 2K2N makes up 19% of the population, which is still greater than NIF 15 at 48 hours at 14% of the population. Nonetheless the reviewers’ point is taken, and the line has been reworded to say “general trend” rather than “progressive decrease” now line 328.

Reviewer 1 – “Nonetheless, this study only did analyses on DNA incorporation and cell cycle analyses (and some on ROS). To increase the significance of the study, it may be critical to do more comprehensive analyses of the drug effect on cell cycle using synchronized parasites. There are a couple of methods proposed for cell cycle synchronization in Trypanosoma brucei. Is there any reason not introducing the methodology into this study?”

- While there are two potential methods of cell synchronization available for T. brucei, one has proven to be so toxic to the cells that it is not useful here (hydroxyurea) and the other centrifugal counter-flow elutriation requires a specific piece of equipment that was not available to us during this research. It is also not considered the required standard in the comparison of cell cycle conditions in trypanosomes. There are many published references for T. brucei cell cycle analysis and comparisons that do not use or require cell synchronization to demonstrate their effects. Cell synchronization may prove critical in future studies aimed at more mechanistic statements. Here we have shown, with asynchronous populations and robust statistical significance that: 1) Fexinidazole decreases the size of the S phase population (FIG. 2 and 3), 2) Fexinidazole decreases the number of cells undergoing DNA synthesis in the population (FIG. 4), and 3) Fexinidazole increases the amount of DNA damage arising between S phase and G2 cell populations. We think that is sufficient to say something new and important about fexinidazole induced cytotoxicity and hope that Reviewer 1 will agree. Future mechanistic studies will utilize cell synchronization.

Reviewer 1 – “L338: Why is phleomycin abbreviated as BLE?”

- We apologize for this error. The selectable marker for phleomycin is the BLE gene and this was the basis of the error. BLE has been changed to “PH” throughout the manuscript, figures, figure legends.

**Response to Reviewer 3**

Reviewer #3: The authors address a critical and recent topic regarding HAT treatment, demonstrating in vitro the possible mechanisms of action of three drugs, focusing on fexinidazole, a drug approved by DNDi for the oral treatment of African trypanosomiasis. The text and results are clear, corroborating most of the proposed findings. However, there are some improvements that could adapt the study to the journal format and facilitate the understanding of the manuscript.

We appreciate the summary by Rev 2 noting that the text and results are clear and corroborate most of the proposed finding and will endeavor to improve the manuscript in response to their suggestions.

Suggestions:
1) Manuscript organization: I suggest that authors review the “manuscript organization” section as authors of research articles are asked to add an Author summary after the Abstract.
Thank you, we have added an “Authors Summary” after the abstract: lines 97-107.

- I suggest that authors review the format requested by the journal for figure labels and in-text citations throughout the text and in the figure captions.
Thank you, we have reviewed the journals formatting guidelines and endeavored to better adhere to them throughout the manuscript.

2) Introduction:
The introduction is clear and objective, but I suggest the authors discuss African trypanosomiasis in more detail, providing recent data on its epidemiology, number of cases, and endemic regions, since the article's focus seems to be more related to HAT than to other trypanosomiasis.
Line 113: Add a reference to the text about HAT.

Added in lines 122-125.

3) Results:
Lines 193-202: In the excerpt, the authors use the terms "low" and "high" to discuss the concentrations of nitroaromatic drugs. However, it's unclear whether when they refer to all drug concentrations (low and high), they are referring to Figure 1B or 1C (or both). It's in Figure C that the authors show the graphs of low nitro compared and high nitro compared (and cite in the figure caption as "more detailed visualization of data show in panel (B)"). I suggest the authors review this information and, if they find it pertinent, add to the excerpt that the results presented are in Figures 1B-C.
We apologize for any confusion, the HIGH & LOW drug concentrations established in FIG. 1 are defined in lines 307 & 310, FIG.1, and the figure legend. Specific concentrations used are now listed in all figures or figure legends.

Lines 246-251: I suggest the authors add a reference at the end of the paragraph.

References have been added to lines 368, 373, & 374.

Fig 6: The authors show the overlay of the different drugs in the last graphs of Figure 6A, but I believe an overlay using a contour plot would be more visual and highlight the results.

We agree and have made this change.

Lines 366-367:
- I suggest the authors add to the legend of Fig. 6 the statistical analysis used in graph 6C (t-test, one-way or two-way ANOVA, or others).

All statistical details have been added to the methods.
Fig 6D: Is there any reason the authors used flow cytometry at 3, 6, and 12 hours and Western blot at 3, 6, 12, and 24 hours? Do the authors expect that the DNA damage induced by the drugs, especially fexinidazole, would be greater at 24 hours?

There were technical reasons why 24 hours was not analyzed by flow cytometry. To avoid the 24 hour timepoint was removed from the western.

Line 375: "... (FIG. 7B, note the maximum scale of graph G1)." I suggest the authors review whether the journal allows this type of configuration for in-text citations.

It is our understanding that it is acceptable, and we highlighted the difference in scale to avoid misinterpretation of the data.

Fig 7: I suggest the authors add in the legend the statistical analysis used in graphs A and C (t-test, one-way or two-way anova, or others).
We have added a detailed summary of our statistical analyses for all flow data to the methods as an independent section named: **Statistical analysis of flow cytometry data.** In addition, the new supplemental table show all of the raw data and their statistical derivations.

- The authors, based on yH2A-Phos and DAPI staining, quantified the proportion of DNA damage in each phase of the cell cycle, adding multinucleated cells to the last quadrant. Why evaluate these cells? Would this evaluation indicate that the drug was interfering with cell cycle progression? Is there a statistical difference from this data in Figure 7C (Multinuclear damage)?

FIG. 7 has been changed such that 7B shows the % DNA damage vs time for each drug with a graph for each stage of cell cycle followed by 7C showing the drugs in G1, S, and G2.

Why show multinucleated cells: MN cells often arise as an outcome of DNA damage or other DNA metabolism insult. Rather than halting nuclei division as mammalian cells do in response to DNA damage, trypanosomes often proceed to divide nuclei but then do not undergo cytokinesis and MN cells form. Thus, it is a population of interest in this consideration. It is not included in the bar graphs of FIG. 7C because the figure was already rather busy and it was eliminated for simplicity.

- I suggest the authors add the yH2A-Phos and DAPI subgates with the NIF, BENZ, and FEX treatments to complement the data shown in Fig 7C (Multinuclear DNA damage).

FIG. 7A now shows representative data from all 3 drugs and SUP. 15 shows all gates, raw counts, and percentages from 2 replicates of each drug with all raw data available in SUP 16.

4) Methods:
Lines 495-528: I suggest authors review this Methods section, as despite containing all the relevant information, the text is lengthy and somewhat confusing. I suggest dividing it into more sections could improve reader understanding.
Line 498: The authors cite the drug eflornithine, but it is not mentioned throughout the paper, only in the discussion because it is a combination therapy with nifurtimox in humans. Was this drug used? If not, I suggest the authors remove it from the Methods.

The methods have been revised to increase clarity and reduce redundancy. Eflornithine has been removed from the methods.

Lines 530-538: I suggest that the authors remove the statistical information from this topic and add a new topic “Statistical analysis” so that they can add information on which statistical analyses were used, which tool (e.g. Graph Pad Prism) and which p value was considered statistically significant.
Line 535: I suggest that the authors add which version of FlowJo was used.

Thank you we have added this section to the methods.

Line 542: Since this is the first time the authors have cited "PBS," I suggest writing out the solution name.
Done

Lines 555-556: I suggest the authors add the centrifugation speed (rpm).
Done

5) References:
Line 845: I suggest that the authors review the reference, as it was repeated in (1) (line 827).

Done

Regarding Rev 2 commentary covered in the subsections associated with “Key Review Criteria Required for Acceptance”

-Are the objectives of the study clearly articulated with a clear testable hypothesis stated?

Reviewer #2: 1. Are the objectives of the study clearly articulated with a clear testable hypothesis stated?
The overall objective of the manuscript is relevant and timely—namely, to delineate the specific cytotoxic effects of fexinidazole in T. brucei and to distinguish these from other nitroaromatic drugs. However, the hypothesis that "fexinidazole-induced DNA damage precedes and drives ROS formation and DNA synthesis inhibition" is only partially supported by the current data and not fully testable in its present form.

As mentioned above, it was not our intention to state that hypothesis. We apologize for any confusion on this point and have sought to clarify this matter throughout the revision as described above.

However, upon further consideration we decided to clarify the purpose of the study in the introduction in the following, lines 148-150: “Here we evaluate the proposal that nitroaromatic drugs kill trypanosomes through ROS activation of DNA damage by comparing the trypanocidal outcomes of fexinidazole, nifurtimox, and benznidazole.”

We remain hopeful that these changes are sufficiently conservative while clarifying the theoretical basis of the research.

Required Revision:
To address the mechanistic objective of the study, additional assays are needed to define the nature of DNA damage (e.g., comet assay, adduct detection) or repair activity. Alternatively, the authors must adjust the scope and interpretations of their claims to align with their descriptive findings.
3. Is the population clearly described and appropriate for the hypothesis being tested?
Yes. The use of Trypanosoma brucei bloodstream form (Lister427, SM line) is appropriate and well-justified for studying fexinidazole’s effects in the context of HAT.
4. Is the sample size sufficient to ensure adequate power to address the hypothesis being tested?
The study claims to use three or more biological replicates in most assays. However, statistical analysis details are limited, and it's not clear whether power calculations or tests for significance across multiple comparisons were applied correctly.
Required Revision:
Authors should provide detailed information on sample sizes for each experiment, clarify whether replicates were technical or biological, specify the statistical tests used, and apply corrections for multiple comparisons where appropriate.
5. Were correct statistical analyses used to support conclusions?
Statistical significance is reported, but the manuscript lacks sufficient detail regarding the tests used, assumptions checked (e.g., normality), and whether any multiple hypothesis correction was applied.
Required Revision:
The authors must clearly describe statistical methods in both the figure legends and the Methods section, including assumptions, test types, software used, and correction methods for multiple comparisons.
6. Are there concerns about ethical or regulatory requirements being met?
No concerns are noted. The study involves in vitro parasite culture only, with no vertebrate or human subjects. Ethical standards appear to have been met.
Summary
While the study provides valuable comparative insights into nitroaromatic drug effects in T. brucei, it falls short of providing mechanistic clarity sufficient for publication without substantial revision. Addressing the experimental and interpretive limitations detailed above is required for acceptance.

Please review the changes made associated with section 1 and 2 of this response to see our efforts to rectify these concerns.

Reviewer #2: 1. Does the analysis presented match the analysis plan?
Yes, the analyses align with the general objectives stated in the Introduction. The authors aimed to compare the effects of fexinidazole, benznidazole, and nifurtimox on T. brucei cell cycle progression, DNA synthesis, DNA damage, and ROS production. The study follows a logical flow of experiments from drug titration and viability assays to mechanistic phenotyping.
However, given the stated goal to uncover drug-specific mechanisms, the current analyses remain largely descriptive. The mechanistic implications discussed (e.g., DNA damage initiating ROS and DNA synthesis inhibition) are not directly tested or planned for in the methodology, which creates a mismatch between the depth of analysis and the interpretation.
Suggestion: The authors should either (a) temper their mechanistic claims in the discussion, or (b) provide additional experiments to directly test the sequence of events hypothesized.
We hope that the discussion of mechanism and our changes above are sufficient for publication.

2. Are the results clearly and completely presented?
The results are generally well organized and presented in a logical sequence. Each experimental section is supported by descriptive narrative and corresponding figures. However, the following points require attention:
Some key experimental parameters (e.g., exact sample sizes per experiment, gating strategy details, biological vs. technical replicates) are missing or buried in supplementary material;
Statistical results are incompletely reported—many comparisons lack clear p-values, test types, or error bar definitions;
Conclusions occasionally go beyond what the data directly show, especially in attributing causality between phenotypes.
Suggestion: The clarity and rigor of the Results section would benefit from enhanced statistical transparency and more cautious language where mechanistic inference is not directly supported.
We believe that the updated supplemental material and new methods section described above now fulfill this requirement.

3. Are the figures (Tables, Images) of sufficient quality for clarity?
Figures are generally of good technical quality and include appropriate biological replicates. Flow cytometry plots, histograms, and violin plots are effectively used to convey quantitative data. However:
Some flow cytometry figures (e.g., Figures 6–7) would benefit from more explicit axis labeling and gating overlays;
Figure legends should include full definitions of abbreviations, concentrations, and the number of replicates;

We believe that the updated figures and supplemental material above now fulfill this requirement.

Figure 8 (mechanistic model) should more clearly indicate which pathways are data-driven vs. speculative.

We hope that the figure and figure legend modifications along with the modified manuscript text listed above fulfill this requirement.

Reviewer #2: 1. Are the conclusions supported by the data presented?
The authors conclude that fexinidazole exerts a distinct trypanocidal effect through early and pronounced inhibition of DNA synthesis, along with DNA damage accumulation and ROS formation. While their experimental data clearly show differences in cell cycle profiles, DNA synthesis (via BrdU), and γH2A phosphorylation compared to other nitroaromatic drugs, the claim of a unique mechanistic sequence—where DNA damage precedes and drives the other effects—is only partially supported.
In particular, the evidence does not definitively establish causality or sequence between DNA damage, ROS, and DNA synthesis inhibition. The timepoints measured for ROS and DNA damage differ in resolution, and the mechanism of damage (e.g., oxidative vs. alkylation) is not defined. Therefore, certain conclusions are overstated relative to the data.
Recommendation: The authors should temper their mechanistic claims and avoid inferring causality without direct supporting experiments.

We have endeavored to satisfactorily address this concern in the revised manuscript.

2. Are the limitations of analysis clearly described?
The manuscript acknowledges certain limitations, including the lack of knowledge regarding the chemical nature of fexinidazole’s active metabolites and the possibility of cross-resistance via NTR mutations. However, important analytical limitations are not fully addressed, such as:
The inability to detect early ROS events due to assay limitations;
The lack of specificity of γH2A as a DNA damage marker;
The absence of DNA repair analysis or metabolite profiling.
Recommendation: The authors should expand the discussion of limitations to provide a more balanced perspective and clarify where further work is needed.

Added to discussion lines 582-590.

3. Do the authors discuss how these data can be helpful to advance our understanding of the topic under study?
Yes. The authors correctly highlight the need for better understanding of nitroaromatic drug mechanisms, especially for fexinidazole, which is now a first-line oral treatment for HAT. They point out that DNA synthesis inhibition could represent a key vulnerability in T. brucei, and that differentiating the cytotoxic effects of individual drugs may inform future drug design.
This is a useful and important perspective, particularly given the limited pipeline for kinetoplastid-targeting therapeutics and growing concerns about drug resistance.

Rev 2 remarks are greatly appreciated.

4. Is public health relevance addressed?
Yes, the clinical importance of fexinidazole is discussed in the context of replacing melarsoprol, a highly toxic arsenical drug, for second-stage HAT. The manuscript also notes the potential of fexinidazole for broader use against other kinetoplastids, including Leishmania and T. cruzi. These points are appropriately highlighted in the Introduction and Discussion.
Rev 2 remarks are greatly appreciated.

That said, the link between the current molecular findings and broader public health implications could be made stronger. For example, how might these mechanistic insights inform drug resistance monitoring in endemic settings? Can DNA damage be used as a biomarker of treatment efficacy or toxicity?

This is an interesting consideration that we will make use of in the future.

1. Language and Style:
Some sections of the Discussion include speculative or overly definitive statements (e.g., "DNA damage precedes and drives ROS and DNA synthesis inhibition"). These should be revised for greater caution and scientific neutrality (e.g., “may contribute to” or “is temporally associated with”).
Avoid redundancy in phrase structures (e.g., “the results show that fexinidazole treatment results in…” could be simplified).
Define abbreviations consistently upon first use (e.g., gH2A-P, BrdU, ROS) and ensure they are used uniformly throughout the text.
Minor grammatical and syntactic edits are needed in some figure legends and method descriptions.
We have endeavored to make changes in keeping with these suggestions.

2. Figure Improvements:
Figures 6 & 7 (Flow Cytometry):
Improve axis labeling (e.g., fluorescence units, DAPI intensity), and clearly mark gating thresholds used to define positive populations.
Include representative dot plots or histograms for key time points, ideally with overlays of untreated and positive control (phleomycin-treated) for visual comparison.
We hope the figure revisions and supplements satisfy these concerns.

Figure 8 (Proposed Model):
Distinguish more clearly between experimentally supported pathways and hypothetical ones. For example, use dashed arrows for proposed links and solid arrows for supported mechanisms. Explicitly label speculative pathways as “putative.”
We hope that the figure and figure legend modifications along with the modified manuscript text listed above fulfill this requirement.

Figure Legends:
Provide full details in legends so that figures are interpretable independently of the main text. This includes specifying drug concentrations, treatment durations, number of replicates, statistical methods (e.g., “mean ± SD from n=3 biological replicates”), and significance thresholds.

These details were more clearly addressed in the newly added methods section. We hope this is acceptable.

3. Data Clarity and Statistics:
Specify in the text and figure legends whether error bars represent standard deviation (SD) or standard error of the mean (SEM).
Clarify the statistical tests used for each dataset, including whether multiple comparisons were adjusted and whether data distribution assumptions were tested.
Consider presenting selected summary data in tabular form in Supplementary Materials for transparency (e.g., % BrdU-positive cells at each time point for all drugs).
This has been clarified.

4. Supplemental Data:
The manuscript refers to multiple supplemental figures, but it would be helpful to include a supplemental table summarizing EC50, IC90, and final concentrations used for each drug to facilitate direct comparison.

This is now supplement 2.

Consider including raw flow cytometry plots and gating strategies as supplementary figures for reproducibility.

These are now shown in both gating strategy supplements and raw data supplements.

5. Data Availability and Transparency:
The data availability statement should include a DOI or repository link for raw data (e.g., flow cytometry files, western blots), especially for key experiments involving gating and quantification.

To the best of our knowledge there is not currently a good public repository avalible for flow data. The one suggested by PLOS (<http://flowrepository.org>) is no longer accepting new data sets. Here we have included all raw exported data from flowjo in the added data supplements. We will make all data publicly available when an appropriate repository becomes avalible.

Summary:
These editorial and presentation-related suggestions are **intended to improve the clarity, transparency, and visual quality of the manuscript. None of the above issues warrant rejection or invalidate the conclusions but are important for enhancing scientific communication and reproducibility.**
If the authors implement these modifications alongside the more substantive revisions requested, the manuscript will be significantly improved in clarity and presentation.

We deeply appreciate the time and effort the reviewers’ have put into the improvement of this important manuscript. In this response to reviewers’ we have utilized their input to generate a considerable improvement to the manuscript by way of a major revision. Herein we have sought to accomplish the goals of – ***complete data transparency, careful and conservative interpretation of our data, and clear presentation of the most key findings that can benefit the greater research community.***
