# Supplementary material for: Fexinidazole results in specific effects on DNA synthesis and DNA damage in the African trypanosome
Source: PLoS Negl Trop Dis. 2025 Oct 24;19(10):e0013647. doi: 10.1371/journal.pntd.0013647 (PMC12574827; doi:10.1371/journal.pntd.0013647)
Supplement: Attachment — Submitted filename: Response to Reviewers_PNTD-D-25-00887R1.docx [file pntd.0013647.s019.docx]

**Response to Reviewers- PNTD-D-25-00887R1**

**Dear Editors and Reviewers:**

We appreciate the time and investment that you have given in consideration of this manuscript. The specific effects of fexinidazole on trypanosome cytotoxicity have not been previously reported and the findings here in are a significant contribution to the field of NTDs.

In this second revision we seek to take a conservative approach to addressing the reviewer’s comments in a manner that otherwise leaves the manuscript largely unchanged from the previous version. Thus, the “blue marked-up copy” has all of the previous changes converted to black text and only the changes associated with this revised version will be found in blue. Please find our response to the reviewer’s comments below. *Note: In keeping with PLOS NTD Guidelines, the “Clean Copy” has figure legends placed after the associated results section, which changes the line numbers between Blue/marked-up copy and clean copy. Thank you.

**REV1 –**

Figure 2B: It is not appropriate to compare the four groups one-by-one by picking two out of the four groups. The current form has problems by showing the identical data in separate graphs and by choosing inappropriate statistical analyses.

In response to this reviewer’s comment, we have changed the statistical analysis of cell cycle data presented in FIG. 2B such that it shows all statistically significant changes in S phase (Left graph) or G2 (Right graph). Thus, we have shown and highlighted all statistically significant changes during drug treatments in comparison with untreated (UT). In addition, and in keeping with previous comments from other reviewers we included comparison of FEX with other drugs for S phase populations. Thus demonstrating that the reduction in S phase parasite populations is specific to fexinidazole.

Line changes associated with this can be found in the results lines 337-340 & 346 and figure legend of Figure 2.

I asked about decreased 2K1N and increased 2K2N. Because 2K2N follows after 2K1N, if 2K1N is forced to decrease, the following 2K2N should decrease too in a simple model. Another model to explain decreased 2K1N and increased 2K2N would be an accelerated/shortened cycle of 2K1N. Nonetheless, the authors failed to comprehensively discuss their data again.

We disagree that this concern was “not addressed”. As noted in our previous response we discussed the potential source of our observations in the results lines 374-377 and discussion lines 559-576, where we devoted an entire paragraph to the topic. While Rev 1 is correct that in a simple model inhibition of DNA synthesis would result in the loss of G2 cells, each cell cycle analysis can only present a snapshot of the time at which it was taken. Thus, what we see at that time, for instance decrease in S phase and increase in G2, may seem contradictory but do not speak to how the parasites will recover from this stress over time.

In the discussion we present our hypothesis for these outcomes (lines 573-576), that the DNA damage is resulting in the formation of damaged nuclei that result in asymmetric cell division events that result in the formation of an anucleated daughter and an accumulation of aberrant G2 cells that arise from the parent retaining both nuclei. We show as evidence for this the 70 uM treatment with FEX in Fig. 2, which shows and increase in both G2 cells and sub-G1/zoid peak. Thus, no additional manuscript changes were made in response to this comment.

I also pointed out the discrepancy between Figure 2 and Figure 3 in the original article, as the original Figure 2 showed rather decreased G2 phase (2K2N) while Figure 3 showing increased 2K2N. Then the authors just hid the data on G2 phase in Figure 2B. But unfortunately, the flow data in Figure 2A still says the proportion of G2 is rather decreased by FEX treatment and the trend is completely opposite from one shown in Figure 3B.

It was not our intention to disregard this point upon resubmission, and it was covered in the discussion Lines 565-569. To more thoroughly respond to this concern, we have added lines 391-396 to the results.

I also mentioned ‘For example, NIF 48 h has 14% 2K2N (G2), which is higher than that of UT’ and a response from the authors was ‘19% on UT is greater than 14% on NIF 48h’. It is sad that they do not understand the difference between absolute numbers and percentages. The numbers in the bar graph are absolute numbers but not percentages, and the authors should compare percentages, i.e., 19/154 =12% on UT and 14/102 = 14% on NIF.

To address this point and more clearly communicate the data the following changes have been made:

1) SUP 7 now includes the table of percentages arising from absolute numbers shown in the graph on FIG. 3B. Absolute numbers were shown on the graph to clearly indicate the number of cell events include in each percent value, included for maximum data transparency.

2) To address the specific discrepancies in 2K2N decrease upon NIF and BENZ treatment lines 391-395 have been added.

**REV 2 –**

Reviewer #2: This is a well-conceived and carefully executed mechanistic study that fills a clear gap by comparing three frontline nitro drugs—nifurtimox, benznidazole, and fexinidazole—side-by-side in bloodstream Trypanosoma brucei. The work is timely and relevant to human African trypanosomiasis (HAT), and the manuscript is clearly written, logically structured, and easy to follow.
Novelty & significance: A genuine head-to-head evaluation that delineates a distinct functional signature for fexinidazole (relative to nifurtimox/benznidazole), advancing our understanding of nitro-drug action and offering hypotheses for regimen optimization or combinations in HAT.
Sound design & orthogonal readouts: A coherent pipeline (dose–response and growth/death kinetics to define sublethal windows → BrdU for DNA synthesis → γH2A for DNA damage → ROS assays → cell-cycle profiling) with appropriate positive/negative controls and live/dead discrimination, providing convergent mechanistic evidence.
Rigor & reproducibility: Methods are detailed (reagents, instruments, software), gating strategies are transparent, and replicate structure and time-course design enable consistent interpretation across assays.
Presentation quality: Figures are clear and well annotated; the narrative maintains excellent alignment between aims, methods, results, and conclusions, with a balanced discussion that situates the findings in current literature.
Public-health relevance: The conclusions explicitly connect mechanistic differences to therapeutic decision-making in HAT (e.g., informing dosing windows, potential biomarkers, and resistance monitoring).
Ethics/compliance: Entirely in vitro; no concerns regarding research or publication ethics. No evidence of dual publication.

We kindly thank Rev2.

**REV 3 –**

The authors made the suggested corrections, with the exception of those listed below. I suggest reviewing these points to further enhance the quality of the publication.

We kindly thank Rev3 and have made the final changes recommended below.

Figure 6 and 7: I suggest that the authors add which statistical test was used (t-test/anova) in the figure legend.

Descriptions of statistical analyses performed can now be found in the legends of FIG. 6 & 7, as well as the methods.

References: I suggest that authors review the list of references, as ref Deeks ED. Fexinidazole: First Global Approval. Drugs 2019, 79:215-20, remains duplicated on lines 848 and 876.

We have corrected the redundant citation by including a new citation #11, also by Deeks et al, doi: 10.1007/s40267-019-00672-2

**FINAL CHANGES:**

**FIG 2 –** was changed to more clearly illustrate the statistical significance of the effect on NIF and BENZ on G2, the effect of FEX on S phase, and S phase comparison between FEX vs NIF or BENZ.

**SUP 7** – was changed to add a table of the percent of parasites in each cell cycle population for each condition. This was done to clarify the raw cell count vs. percent of total.

Changes were made to the manuscript Text and figure legends, highlighted in blue on marked up copy.

Statistical methods were added to the legends of FIG. 6 & 7.

The erroneous citation was corrected.
